# Extrapulmonary tuberculosis in The Netherlands, an epidemiologic overview, 1993–2022

**DOI:** 10.1016/j.jctube.2025.100546

**Published:** 2025-06-26

**Authors:** Frouke A. Procee, Jizzo R. Bosdriesz, Frank G.J. Cobelens, Maria Prins, Sabine M. Hermans, Anton E. Kunst

**Affiliations:** a Department of Global Health, Amsterdam University Medical Centers, Location University of Amsterdam, Amsterdam, The Netherlands; b Department of Infectious Diseases, Public Health Service of Amsterdam, Amsterdam, The Netherlands; c Department of Infectious Diseases, Division of Internal Medicine, Amsterdam University Medical Centers, Amsterdam, The Netherlands; d Amsterdam Institute for Infection and Immunity, Amsterdam University Medical Centers, Amsterdam, The Netherlands; e Amsterdam Institute for Global Health and Development, Amsterdam, The Netherlands; f Centre for Tropical Medicine and Travel Medicine, Department of Infectious Diseases, Amsterdam University Medical Centers, Amsterdam, The Netherlands; g Department of Public and Occupational Health, Amsterdam University Medical Centers, Amsterdam, The Netherlands

**Keywords:** Extrapulmonary tuberculosis (EPTB), rising proportion of EPTB, TB epidemiology in low-incidence setting, migrant health and TB, TB in women and children, TBI screening

## Abstract

**Background:**

Extrapulmonary tuberculosis (EPTB) poses significant diagnostic and therapeutic challenges in low-incidence settings like the Netherlands. Despite declining overall tuberculosis (TB) incidence, the proportion of EPTB has risen, especially among migrant populations. This study examines sociodemographic, migration-related, and clinical factors associated with EPTB from 1993 to 2022 to inform TB diagnostics and care.

**Methods:**

A retrospective quantitative analysis of 34,048 TB patients reported to the Netherlands Tuberculosis Registry (1993–2022) was conducted. Logistic regression was used to identify associations with EPTB. Temporal trends in EPTB and pulmonary TB (PTB) were evaluated, including stratification by age, country of birth, and duration of residency.

**Results:**

Over the study period, the proportion of EPTB rose from 37 % to 50 %. EPTB was more common in women (adjusted odds ratio (aOR) 1.53; 95 % CI 1.45–1.62) and children under 14 years (aOR 2.83; 95 % CI 2.46–3.24). Foreign-born individuals, particularly from India, Somalia, Eritrea, Ethiopia and Pakistan, had higher odds of EPTB compared to Dutch-born individuals (aOR range: 2.33–3.86). EPTB was also associated with HIV infection (aOR 1.73; 95 % CI 1.43–2.11) but inversely related to social risk factors like homelessness and problem substance use. TB was notably frequently diagnosed among individuals residing in the Netherlands for over 10 years, more often EPTB than PTB.

**Conclusion:**

The rising proportion of EPTB underscores the need for targeted interventions, particularly for high-risk groups such as women, children and migrants. Enhanced screening, early detection, and preventive strategies, especially for tuberculosis infection (TBI) are critical to reducing EPTB morbidity and mortality.

## 1 Introduction

Tuberculosis (TB), a communicable disease caused by *Mycobacterium tuberculosis,* remains a major global public health problem affecting around 10 million new patients annually [1]. The incidence of tuberculosis has surged to its highest levels in recent history, particularly affecting low and lower-middle-income countries following the COVID-19 pandemic [1,2].

Tuberculosis is traditionally divided into pulmonary (PTB) and extrapulmonary tuberculosis (EPTB). While PTB receives greater attention in TB control programmes because of its transmissibility, EPTB presents its own challenges [2]. Of the globally notified TB cases in 2023, 16 % were EPTB [2]. EPTB can affect nearly any organ, with the most common manifestations being lymph node, pleural, urogenital and abdominal TB, miliary forms of TB, and TB of bones and the central nervous system [3,4]. EPTB usually manifests with a wide spectrum of clinical features and therefore causes more diagnostic and management challenges than PTB [5,6]. In well-resourced settings with low prevalence this can be even more difficult because the diagnosis is often not considered. This diagnostic delay can cause greater morbidity and mortality, and can contribute to post-TB sequelae [4,5,6,7].

In several low incidence countries, the relative proportions of PTB and EPTB have been changing with a noticeable increase in the proportion of EPTB patients [8,9,10,11]. This is partly attributed to the higher number of foreign-born TB patients in these countries [8,9,10,12]. Certain ethnic groups appear more susceptible to developing EPTB [13,14,15], and evidence suggests a relationship between ethnicity and the anatomical localisation of EPTB [14,15]. Additionally, specific genotypic lineages of *M. tuberculosis* have been linked to disease manifestation [16,17,18].

Several other determinants associated with EPTB have been identified [13,14,15,19], including immunological factors such as HIV infection and the use of immunosuppressant medication. Additionally, certain populations appear to have an increased risk of EPTB, including women, children, the elderly, and in low incidence countries also people with a migrant background from high endemic regions [13,14,15].

The Netherlands is a low tuberculosis incidence country with rates of 4.1 cases per 100,000 people in 2022. The majority (75 %) of these patients were born in high-endemic countries and there is an increase in EPTB versus PTB as well [20]. TB care and control in The Netherlands is structured with comprehensive national guidelines, including for the screening programmes conducted by the Public Health Services [21]. Additionally, there are guidelines that support healthcare workers in their diagnostic and therapeutic approach to patients with (suspected) tuberculosis [21].

Understanding the characteristics of EPTB patients is crucial to facilitate early detection and to prevent severe complications due to delayed diagnosis, and potential post-TB sequelae. The most recent epidemiological study on EPTB in the Netherlands covered 1993 till 2001 and focussed mainly on the nationality of the affected people [22]. To update and expand the epidemiological knowledge, this study aims to provide a comprehensive overview of patients diagnosed with EPTB in The Netherlands between 1993 and 2022. We assessed migration-related characteristics, socio-demographic factors and social circumstances. We also explored the influence of diminished immune status. Additionally, we compared the characteristics of people with EPTB to those of people with PTB and assessed trends over time.

## 2 Methods

### 2.1 Study design and study population

This retrospective study analyses data from all TB patients reported in the Netherlands between 1993 and 2022. Data were obtained from the Netherlands Tuberculosis Registry (NTR), a national database that includes pseudonymized records of all patients with confirmed or highly suspected TB to one of the twenty-five regional Public Health Services. Reporting is mandatory, and the NTR, that has been kept since 1993, is estimated to include 99 % of all TB patients diagnosed in the country. By the end of 2022, the registry contained data of 34,048 TB patients [23].

PTB was defined as TB of the lung parenchyma, the tracheobronchial tree, or the larynx. EPTB was defined as TB affecting any other organs or anatomical sites. Patients with both PTB and EPTB, and those with disseminated TB were included in the EPTB group for analysis purposes.

### 2.2 Setting

In the Netherlands, TB care and control is coordinated by the Public Health Services in collaboration with hospital specialists. Approximately 75 % of active TB patients are diagnosed after presenting with symptoms to medical professionals in hospitals. The remaining patients are primarily identified through structured screening programmes.

Mandatory entry screening for immigrants from high TB-endemic countries includes chest X-rays for adults, and tuberculosis infection (TBI) testing with a tuberculin skin test (TST), and/or interferon-gamma release assay (IGRA), and symptom checks for those under 18 years of age. Follow-up screening, though not mandatory, is offered to refugees and immigrants from very high-incidence countries. Until 2021, homeless people and prisoners were also screened using chest X-rays. Since 2018, the Public Health Service is transitioning to TBI screening for eligible immigrants and for asylum seeker children under 12 years of age. All individuals diagnosed with TBI are offered preventive treatment.

Data on active TB caused by *M. tuberculosis* complex are collected using standardized national forms and entered into the NTR by trained public health professionals. The registry is centrally managed by the National Institute for Public Health and the Environment (RIVM), which performs regular quality checks and updates to ensure completeness and consistency.

Data on the total number of Dutch and non-Dutch born inhabitants in the Netherlands during part of the study period (1996–2021) were retrieved from the Central Bureau of Statistics (CBS) [24].

### 2.3 Variables

We used several NTR variables to assess their association with the likelihood of having EPTB: sex, age group (≤ 14, 15–24, 25–34, 35–44, 45–64, ≥ 65 years), country of birth, duration of residence in the Netherlands (0-<6 months, 6-<30 months, 2.5-<5 years, 5-<10 years and 10 years or longer), HIV status (positive or negative), use of any immunosuppressive medication, diabetes mellitus and malignancy (all yes or no). Additional dichotomous variables were documented contacts of an infectious TB patient, homelessness, harmful alcohol use, problem drug use, detention, healthcare work, travel to highly endemic regions (see Table 1 for definitions). Finally, year of diagnosis was considered as a continuous variable.

**Table 1:** Descriptive characteristics of tuberculosis (TB) patients notified in the Netherlands, 1993–2022.

	Total tuberculosis (TB)		Extrapulmonary tuberculosis (EPTB)		Pulmonary tuberculosis (PTB)	
Characteristics	n	% (column)	n	% (row)	n	% (row)
Total TB	34,048	100	17,666	51.9	16,382	48.1
Sex						
Male Female	20,038 13,997	58.9 41.1	9447 8211	47.1 58.7	10,591 5786	52.9 41.3
Age groups ≤14						
15–24 25–34 35–44 45–64 ≥65	1886 6086 8700 5487 6669 5225	5.5 17.9 25.5 16.1 19.6 15.3	1310 2990 4543 2803 3375 2543	69.5 49.1 52.2 51.1 50.6 48.7	576 3096 4157 2684 3294 2682	30.5 50.9 47.8 48.9 49.4 51.3
Residency in NL						
<6 months 6 months-<2,5 yrs 2,5 yrs-<5 yrs 5-<10 yrs ≥10 yrs	3526 4325 2869 2862 6762	15.3 18.7 12.4 12.4 29.3	1118 2531 1875 1770 3814	31.7 58.5 65.4 61.8 56.4	2408 1794 994 1092 2948	68.3 41.5 34.6 38.2 43.6
Immunosuppression						
Medication (any)	726	2.1	396	54.5	330	45.5
HIV infected	1266	3.7	715	56.5	551	43.5
Diabetes mellitus	1336	3.9	639	47.8	697	52.2
Malignancy	778	2.3	353	45.4	425	54.6
Country of birth						
India Somalia Pakistan Ethiopia Eritrea Afghanistan Suriname Indonesia Morocco Netherlands Turkey Poland	763 3910 559 529 1017 578 1280 1283 3088 10,959 1351 293	3.0 15.3 2.2 2.1 4.0 2.3 5.0 5.0 12.1 42.8 5.3 1.1	553 2808 399 342 623 346 737 728 1659 5037 619 56	72.5 71.8 71.4 64.7 61.3 59.9 57.6 56.7 53.7 46.0 45.8 19.1	210 1102 160 187 394 232 543 555 1429 5922 732 237	27.5 28.2 28.6 35.3 38.7 40.1 42.4 43.3 46.3 54.0 54.2 80.9
Social risk factor						
Contact of infectious TB	3076	9.0	1534	49.9	1542	50.1
Homelessness*	635	1.9	174	27.4	461	72.6
Harmful alcohol use#	469	1.4	98	20.9	371	79.1
Problem drug use#	858	2.5	191	22.3	667	77.7
Detention+	700	2.1	114	16.3	586	83.7
Healthcare worker^	488	1.4	249	51.0	239	49.0
Traveller∼	832	2.4	381	45.8	451	54.2

Column percentages refer to the proportion of the total TB population, row percentages reflect within-group distributions.

HIV: human immunodeficiency virus.

*Homelessness: the absence of a permanent place of residence, as a result of which someone regularly sleeps on the street and/or uses marginal temporary housing or shelters at the time of the first examination or at the time of diagnosis.

# Harmful alcohol use or problem drug use: the regular use of alcohol or hard drugs (including methadone and cocaine), which has led to some kind of social derailment, at the time of first examination or at the time of diagnosis.

+ Detention: the stay in a penitentiary institution (PI) at the time of first examination or at time of diagnosis. This should also include those for whom screening tests are carried out following a stay in the PI, but for whom the diagnosis is only made after discharge from detention.

^ Healthcare worker: a person who by virtue of his professional practice or through volunteer activities, has an increased chance of coming into (intensive) personal contact with untreated TB patients.

∼ Traveller: a person who has spent a total of more than 3 months in the past 2 years in areas where tuberculosis is endemic (prevalence higher than 100/100,000).

### 2.4 Data completeness and analysis

While the NTR captures nearly all notified TB patients nationally, not all records are complete for every variable. Variables with high levels of missing data or inconsistent reporting across the study period were not used for analysis. For included variables, patients with missing key information were excluded from the relevant analyses. For the regression analyses, a subset of patients with complete data and eligible country of birth was used.

We calculated frequencies of EPTB and PTB by year of diagnosis and summarized the demographic characteristics of both EPTB and PTB patients regarding the aforementioned variables.

Univariate and multivariate logistic regression analyses were conducted to identify factors associated with EPTB versus PTB. Covariates included sex, age group, country of birth, comorbidities and social risk factors. Results are presented as (adjusted) odds ratios ((a)OR) and 95 % confidence intervals (95 % CI). Temporal trends were analysed using aggregated data by year. These analyses were stratified by age group and duration of residency in The Netherlands for patients with EPTB. For the 12 countries of birth with the highest numbers of TB patients reported to the NTR, we retrieved the total number of inhabitants in the Netherlands per year (1996–2021) from the CBS data. With the number of reported patients in each corresponding group, we calculated incidences per 100,000 persons of both EPTB and PTB in those years.

Data were analysed using RStudio version 4.3.2 and Microsoft Excel 365.

### 2.5 Ethics

The medical ethics review committee of the Amsterdam University Medical Centre waived the requirement of ethical approval in accordance with the Dutch Act for Medical Research involving Human Subjects (WMO).

## 3 Results

### 3.1 Study population

Between 1993 and 2022, 34,048 people with tuberculosis were reported to the NTR. Of these, 17,666 (51.9 %) were diagnosed with EPTB, including 12.8 % who had both EPTB and PTB. For the regression analyses, 23,253 patients were included. Exclusions were primarily due to country of birth not being among the 12 selected countries, and to a lesser extent, missing data on key variables in the final analysis (see Supplementary Fig. S1).

Demographic characteristics are summarized in Table 1. Compared to PTB, EPTB was more frequently diagnosed in women and in children 14 years or younger (Supplementary Table S1). People born outside the Netherlands, especially those born in India, Somalia, Pakistan, Ethiopia and Eritrea showed higher proportions of EPTB (Table S2). People with HIV were more commonly diagnosed with EPTB, while people with diabetes were more often diagnosed with PTB. PTB was also more frequently observed in people with harmful alcohol use, problem drug use, and homelessness, and particularly among individuals from Poland compared to other countries of birth (Table 1, Table 2).

**Table 2:** Social risk factor to country of birth of tuberculosis patients notified in the Netherlands, 1993–2022.

Country of birth	Homelessness*		Harmful alcohol use#		Problem drug use#		Contact of an infectious TB patient	
	n	%	n	%	n	%	n	%
Netherlands	183	1.7	254	2.4	356	3.4	1893	20.9
Eritrea	10	1.0	2	0.2	6	0.6	89	9.7
Somalia	51	1.3	27	0.7	22	0.6	193	5.2
India	9	1.2	6	0.8	0	0	15	2.0
Ethiopia	6	1.2	2	0.4	3	0.6	14	2.7
Pakistan	4	0.7	1	0.2	0	0	22	4.1
Afghanistan	2	0.3	1	0.2	1	0.2	15	2.7
Indonesia	10	0.8	5	0.4	10	0.8	33	2.6
Morocco	54	1.8	8	0.3	67	2.2	192	6.7
Poland	27	10.5	34	13.5	10	3.6	35	14.0
Turkey	14	1.0	8	0.6	18	1.4	56	4.3
Suriname	53	4.3	29	2.3	138	12.1	114	9.8

*Homelessness: the absence of a permanent place of residence, as a result of which someone regularly sleeps on the street and/or uses marginal temporary housing or shelters at the time of the first examination or at the time of diagnosis.

# Harmful alcohol use or problem drug use: the regular use of alcohol or hard drugs (including methadone and cocaine), which has led to some kind of social derailment, at the time of first examination or at the time of diagnosis.

### 3.2 Comparing EPTB with PTB patients

In multivariate logistic regression analyses (Table 3), the odds of developing EPTB were higher in women compared to men (adjusted odds ratio (aOR) 1.53; 95 % CI 1.45–1.62) and in children younger than 14 years old compared to those aged 25–34 years (aOR 2.83; 95 % CI 2.46–3.24). Conversely, people 65 and older (aOR 0.91; 95 % CI 0.83–1.00) had slightly lower odds of developing EPTB compared to 25–34-year olds.

**Table 3:** Uni-and multivariate regression models of characteristics of tuberculosis (TB) patients notified in the Netherlands by extrapulmonary tuberculosis versus pulmonary tuberculosis, 1993–2022.

	Univariate	Multivariate
Characteristics	OR of having EPTB (95 %CI)	adjusted OR of having EPTB (95 %CI)
Sex		
Male Female	Reference 1.59 (1.52–1.66)	Reference 1.53 (1.45–1.62)
Age groups ≤ 14		
15–24 25–34 35–44 45–64 ≥65	2.13 (1.91–2.37) 0.89 (0.83–0.95) Reference 0.95 (0.89–1.02) 0.93 (0.87–0.99) 0.86 (0.80–0.92)	2.83 (2.46–3.24) 0.90 (0.82–0.98) Reference 0.95 (0.87–1.04) 0.95 (0.87–1.04) 0.91 (0.83–1.00)
Time in NL Born in NL <6 months		
6 months-<2,5 yrs 2,5 yrs-<5 yrs 5-<10 yrs ≥10 yrs	Reference 0.55 (0.51–0.60) 1.68 (1.57–1.80) 2.25 (2.07–2.46) 1.92 (1.76–2.09) 1.53 (1.44–1.63)	Reference 0.28 (0.24–0.32) 0.77 (0.67–0.89) 1.11 (0.95–1.30) 0.95 (0.82–1.11) 0.94 (0.83–1.06)
Immunosuppression		
Medication	1.12 (0.97–1.30)	0.92 (0.78–1.09)
HIV infected	1.21 (1.08–1.36)	1.73 (1.43–2.11)
Diabetes	0.85 (0.76–0.94)	0.83 (0.73–0.95)
Malignancy	0.77 (0.66–0.88)	0.87 (0.74–1.03)
Country of birth Netherlands		
India Somalia Pakistan Eritrea Ethiopia Suriname Afghanistan Indonesia Morocco Turkey Poland	Reference 2.64 (2.19–3.18) 2.55 (2.20–2.95) 2.49 (2.09–2.98) 1.58 (1.22–2.05) 1.83 (1.47–2.27) 1,36 (1,19–1,54) 1.49 (1.27–1.76) 1.31 (1.17–1.47) 1.16 (1.10–1.23) 0.85 (0.76–0.95) 0.24 (0.18–0.31)	Reference 3.86 (3.14–4.77) 3.85 (3.34–4.43) 3.72 (2.98–4.68) 2.44 (2.01–2.96) 2.33 (1.86–2.92) 2.09 (1.78–2.46) 2.04 (1.65–2.52) 1.75 (1.49–2.06) 1.59 (1.39–1.82) 1.17 (1.00–1.37) 0.32 (0.22–0.44)
Year of diagnosis	1.02 (1.01–1.02)	1.02 (1.01–1.02)
Social risk factor		
Contact of documented TB	0.91 (0.85–0.99)	0.72 (0.65–0.79)
Homelessness*	0.34 (0.29–0.41)	0.50 (0.39–0.64)
Harmful alcohol use#	0.24 (0.19–0.30)	0.40 (0.30–0.51)
Problem drug use#	0.26 (0.22–0.30)	0.32 (0.26–0.40)
Detention+	0.18 (0.14–0.21)	0.32 (0.24–0.41)
Healthcare worker^	0.97 (0.81–1.16)	1.02 (0.84–1.25)
Traveller∼	0.78 (0.68–0.89)	0.80 (0.68–0.95)

OR: Odds Ratio, aOR: adjusted OR, 95% CI: 95% Confidence Interval.

HIV: human immunodeficiency virus.

*Homelessness: the absence of a permanent place of residence, as a result of which someone regularly sleeps on the street and/or uses marginal temporary housing or shelters at the time of the first examination or at the time of diagnosis.

# Harmful alcohol use or problem drug use: the regular use of alcohol or hard drugs (including methadone and cocaine), which has led to some kind of social derailment, at the time of first examination or at the time of diagnosis.

+ Detention: the stay in a penitentiary institution (PI) at the time of first examination or at time of diagnosis. This should also include those for whom screening tests are carried out following a stay in the PI, but for whom the diagnosis is only made after discharge from detention.

^ Healthcare worker: a person who by virtue of his professional practice or through volunteer activities, has an increased chance of coming into (intensive) personal contact with untreated TB patients.

∼ Traveller: a person who has spent a total of more than 3 months in the past 2 years in areas where tuberculosis is endemic (prevalence higher than 100/100,000).

Compared to individuals born in the Netherlands, most foreign-born individuals had significantly higher odds of developing EPTB, with the strongest associations observed in people born in India (aOR 3.86; 95 % CI 3.14–4.77), Somalia (aOR 3.85; 95 % CI 3.34–4.43), Pakistan, Eritrea and Ethiopia. In contrast, people born in Poland had significantly lower odds (aOR 0.32; 95 % CI 0.22–0.44) of being diagnosed with EPTB.

Moreover, in this multivariate regression analysis, newcomers with less than 6 months residency in the Netherlands (aOR 0.28; 95 % CI 0.24–0.32), and between 6 months and 2.5 years residency (aOR 0.77; 95 % CI 0.67–0.89) had lower odds of having EPTB compared to people born in the Netherlands. The finding in the univariate regression analysis (Table 2) with significantly higher odds in the groups living in the Netherlands for 2.5-<5 years, 5-<10 years and 10 years or longer did not hold in the multivariate regression analysis.

Additionally, people living with HIV had higher odds of developing EPTB compared to HIV negative individuals (aOR 1.73; 95 % CI 1.43–2.11). On the other hand, individuals with diabetes had lower odds of being diagnosed with EPTB (aOR 0.83; 95 % CI 0.73–0.95).

Social factors were also significantly associated with lower odds of EPTB. Homelessness (aOR 0.50; 95 % CI 0.39–0.64), harmful alcohol use (aOR 0.40; 95 % CI 0.30–0.51), problem drug use (aOR 0.32; 95 % CI 0.26–0.40), and detention (aOR 0.32; 95 % CI 0.24–0.41) all had reduced odds of being diagnosed with EPTB. Similarly, travellers (aOR 0.80; 95 % CI 0.68–0.95) and contacts of known TB patients (aOR 0.72; 95 % CI 0.65–0.79) had lower odds of having EPTB. Over time, the odds of being diagnosed with EPTB increased slightly each year (aOR 1.02; 95 % CI 1.01–1.02).

### 3.3 Trends over the years

**Fig. 1:**
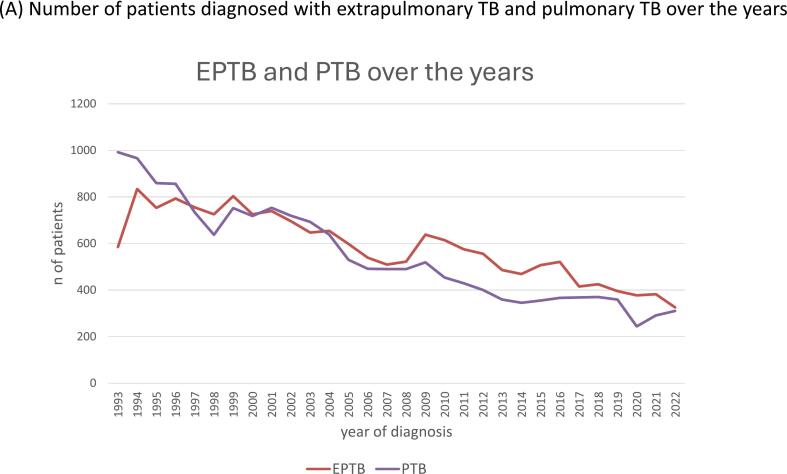

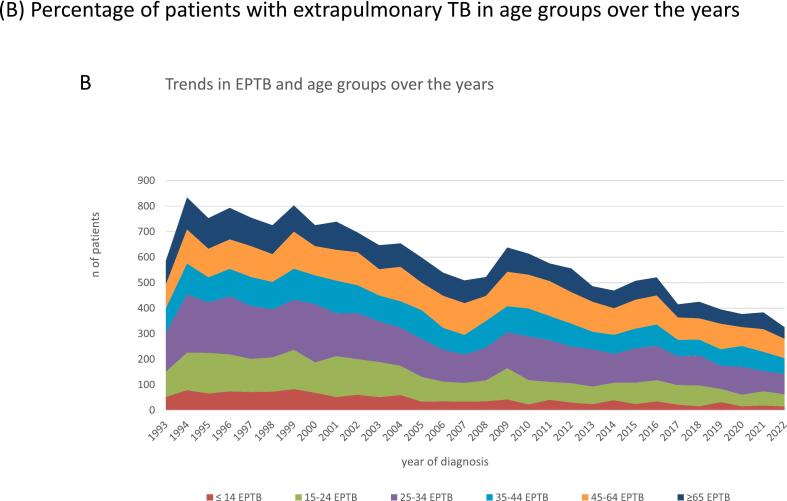

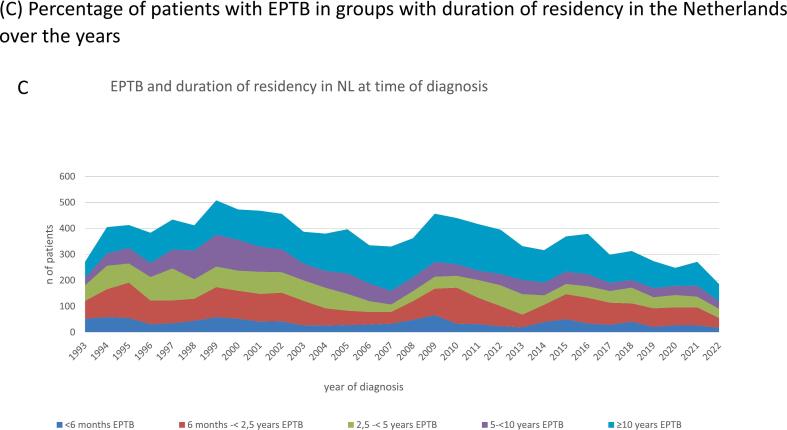

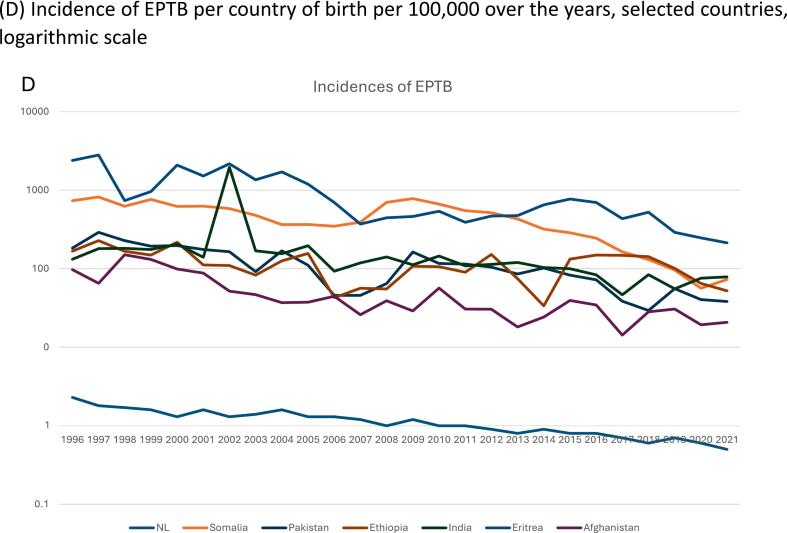
**Trends over time in extrapulmonary tuberculosis (EPTB), The Netherlands, 1993**–**2022.** (A) Number of patients diagnosed with extrapulmonary TB and pulmonary TB over the years. (B) Percentage of patients with extrapulmonary TB in age groups over the years. (C) Percentage of patients with EPTB in groups with duration of residency in the Netherlands over the years. (D) Incidence of EPTB per country of birth per 100,000 over the years, selected countries, logarithmic scale.

While numbers of TB diagnoses gradually declined, the proportion of EPTB rose from 37 % in 1993 to 50 % in 2022 (Fig. 1A). Over the years the number of EPTB patients decreased in all age groups, though with some fluctuations across the years (Fig. 1B). The age group 25–34 years consistently showed higher numbers of patients with EPTB compared to other age groups.

Recently arrived immigrants in the Netherlands were more often diagnosed with PTB (68.3 %). However, EPTB was more frequent after having lived in The Netherlands between 6 months-<2.5 years (58.5 %) or between 2.5-<5 years (65.4 %) (Fig. 1C). Many patients (29.3 %) were still diagnosed with TB after having lived in The Netherlands for more than ten years, more often EPTB (56.4 %) than PTB (43.6 %) (Table 1).

Using demographic data on the influx of specific groups of immigrants, the highest incidences of EPTB were observed in people born in Eritrea, India, Somalia, Ethiopia, and Pakistan (Tables S3–S5). Although there was a declining trend in overall EPTB cases over the years, these five countries consistently had the highest EPTB-to-population ratios (Fig. 1D). This aligns with the associations seen in the regression analysis.

## 4 Discussion

This study describes an analysis of the demographic characteristics and trends associated with EPTB compared to PTB in the Netherlands over almost thirty years. While the overall number of TB patients declined, the proportion of EPTB steadily increased over the years. We found EPTB more often among women and younger age groups when compared to PTB. EPTB was also notably higher in foreign-born people, especially among those from Somalia, India, Pakistan, Ethiopia, and Eritrea, and in people living with HIV. In contrast, PTB was more strongly associated with diabetes and social circumstances like substance use and homelessness. Immigrants residing in the Netherlands for over ten years were more frequently diagnosed with TB than shorter-term residents. In the latter group, the share of EPTB among all TB patients is relatively large.

### 4.1 Rising proportion of EPTB

A key finding is the increasing proportion of EPTB, accounting for 50 % of the TB patients in 2022, despite an overall decline in the total number of TB diagnoses. This proportional increase likely reflects both a sharper decline in PTB and improved detection of EPTB over time (Fig. 1A). This trend, also observed in other TB low-incidence settings [8,9,10,11,12], has several interconnected contributing factors. Firstly, the increase in foreign born patients. Over the years, there has been influx of migrants that show far higher rates of EPTB compared to Dutch-born patients. EPTB is thought to result from the progression of tuberculosis infection (TBI). Most migrants are infected before arriving, either in their country of birth, or during their journey to The Netherlands. Fig. 1D illustrates the fluctuation of incidences due to influx of immigrants because of various geopolitical and employment factors (Table S3).

Additionally, genetic and biological factors of both host and pathogen contribute to the observed trend. Specific *M. tuberculosis* lineages have been linked to phenotypic disease manifestations, such as the East African Indian lineage and peripheral lymph node tuberculosis [16,17,18]. These interactions may partly explain the higher EPTB rates observed in people from Eritrea, Somalia, India, Pakistan and Ethiopia, with rates up to 281 times higher than in Dutch nationals in most recent years. Lastly, differences between migrant populations further shape the trend. The proportional rise in EPTB could partly be attributed to the increased number of highly skilled immigrants from the Indian subcontinent, a group with a notable burden of EPTB. Conversely, Polish nationals, who make up a substantial portion of recent European immigrants, show a lower proportion of EPTB, which aligns with trends observed in Poland [25] and other Eastern European countries [9].

### 4.2 Sex differences and EPTB

In our study women had significantly higher odds of EPTB versus PTB compared to men, consistent with findings from other studies [26,27,28,29]. We also observed that women born in countries with higher EPTB rates such as Eritrea, Somalia and India, continue to have higher EPTB rates than men from the same regions. In contrast, studies on PTB consistently report a higher prevalence among men than among women relative to the total number of TB patients [30], a trend that is only partially explained by differences in exposure, risk factors and access to care [31,32]. These findings underscore the complex role of sex in the pathogenesis of tuberculosis and suggest that further research into biological factors such as hormonal influences and immune responses, alongside social determinants, is essential to fully understand the underlying mechanisms.

### 4.3 TB among migrants

While our descriptive analysis and univariate regressions indicated that among TB patients, long-term immigrants were more likely to develop EPTB, compared to recent arrivals, this association became weaker and was not statistically significant in the multivariate regression analysis. This suggests that other factors, such as age or country of birth, may play a more direct role in influencing EPTB risk.

Among newcomers, TB patients were more often diagnosed with PTB, likely due to the mandatory chest X-ray screening upon arrival in the Netherlands. However, a substantial number of patients, particularly those with EPTB, were diagnosed after residing in The Netherlands for over ten years. This may reflect cumulative reactivation risk, delayed diagnosis of non-acute symptoms, or age-related immune changes, and could also be influenced by evolving healthcare-seeking behavior or increasing use of immunosuppressive therapies. This trend was described before and highlights the need for sustained monitoring and intervention [33]. To address this, the Public Health Service is rolling out programmes to screen refugees and immigrants from very high-incidence countries for TBI and offer them preventive treatment when positive. These initiatives aim to prevent the progression to active TB and reduce TB transmission.

### 4.4 TB and immune status

Immune status also plays a key role in the development of EPTB. In our study, people living with HIV had significantly higher odds of developing EPTB, consistent with previous findings. Conversely, individuals with diabetes, a known risk factor for PTB [34], had lower odds of EPTB. These findings underscore the importance of host immune functioning in determining disease presentation.

### 4.5 TB and social determinants

People facing social challenges like homelessness, substance use, and detention were more frequently diagnosed with PTB compared to EPTB, consistent with other studies [35,36]. In recent years these people have increasingly originated from other European countries and the majority were men [37]. In our study, people from Poland accounted for 10 % of the homeless TB patients. Many were migrant workers who lost their jobs and consequently their housing.

The higher proportion of PTB in these groups may be largely explained by targeted screening practices. Until 2021, routine screening with chest X-rays was standard practice among homeless people and those in detention. This primarily detects PTB, but not extrapulmonary forms. In addition, classic symptoms of PTB, like cough, may be more easily recognized by health care workers in social shelters or prisons, leading to earlier referral and diagnosis. In contrast, EPTB may be underdiagnosed in these vulnerable populations, because of non-specific or subtle symptoms, which may be overlooked in combination with barriers in access to healthcare or delayed care-seeking.

## 5 Strengths and limitations

This study utilizes a comprehensive national dataset covering over 99 % of all TB patients in the Netherlands over thirty years. The large sample size offers substantial statistical power. Stratified regression analyses supported a nuanced understanding of factors associated with EPTB compared to PTB.

Limitations include variability in data completeness related to accuracy of the reports submitted to the NTR, which may have affected the reliability of findings. Some variables like living in a center for asylum seekers, malnutrition, and delay of diagnosis contained too many missing values to be included in the study. For the regression analysis we included only patients with complete data on the variables used in the model and therefore 10,795 patients were excluded (see Supplementary Fig. S1). Incidences calculated using CBS numbers of migrants, without knowing the detailed composition of the group, can have given a distorted picture. The long study period (1993–2022) covers changes in diagnostic practices, health care practices, and immigration patterns, and therefore temporal trends must be interpreted with caution. For instance, increased use of (PET) CT imaging, GeneXpert testing on solid tissue and immunosuppressive therapies, may have contributed to higher EPTB detection over time.

## 6 Implications for TB care and control

While many of our findings align with previously published studies, some reveal interesting patterns that contribute to our understanding of TB epidemiology in a low incidence setting. These patterns show the need for targeted interventions for specific demographic groups. The start that was made in the screening of immigrants for TBI, and treating them before activating to TB disease is promising in preventing people from developing EPTB. The outcomes should be closely monitored. Our study highlights the importance for increasing awareness among healthcare providers in recognizing the risk factors and diagnostic challenges of EPTB. Particularly in migrants, women, children and people living with HIV they should consider the diverse and non-specific symptoms. Given the increasing proportion of EPTB, it is essential that guidelines and professional education are updated with more attention to EPTB and with a focus on early detection and targeted screening for high-risk groups. Future research on the biological factors, social determinants and treatment outcomes, could help further understand the high rates of TB in these groups and reduce delays in diagnosis, improve treatment outcomes and prevent recurrent cases.

## 7 Contributions

The study was conceptualized and designed by FP, AK, FC, MP and JB. Data were collected by FP, statistical analyses were performed by FP and JB. FP, AK, JB, FC and MP contributed to methodology development. The manuscript was drafted by FP and critically revised by JB, AK, FC, MP and SH. All authors reviewed and approved the final manuscript.

## 8 Ethical statement to manuscript: Extrapulmonary tuberculosis in the Netherlands, an epidemiologic overview of 1993–2022

The medical ethics review committee of the Amsterdam University Medical Centre waived the requirement of ethical approval in accordance with the Dutch Act for Medical Research involving Human Subjects (WMO).

## CRediT authorship contribution statement

**Frouke A. Procee:** Writing – review & editing, Writing – original draft, Software, Methodology, Investigation, Formal analysis, Data curation. **Jizzo R. Bosdriesz:** Conceptualization, Formal analysis, Methodology, Writing – review & editing. **Frank G.J. Cobelens:** Conceptualization, Methodology, Supervision, Writing – review & editing. **Maria Prins:** Conceptualization, Methodology, Supervision, Writing – review & editing. **Sabine M. Hermans:** Writing – review & editing. **Anton E. Kunst:** Conceptualization, Methodology, Supervision, Writing – review & editing.

## Declaration of competing interest

The authors declare that they have no known competing financial interests or personal relationships that could have appeared to influence the work reported in this paper.
