# Supplementary material for: Extrapulmonary tuberculosis in The Netherlands, an epidemiologic overview, 1993–2022
Source: J Clin Tuberc Other Mycobact Dis. 2025 Jun 26;40:100546. doi: 10.1016/j.jctube.2025.100546 (PMC12270629; doi:10.1016/j.jctube.2025.100546)
Supplement: Supplementary Data 1 [file mmc1.docx]

**Supplementary Figures and Tables with Extrapulmonary tuberculosis in The Netherlands, a demographic overview of 1993-2022:**

**Figure S1: Flowchart of patient inclusion of TB patients notified to the Netherlands Tuberculosis Registry (NTR) from 1993-2022 for multivariate regression analysis.**


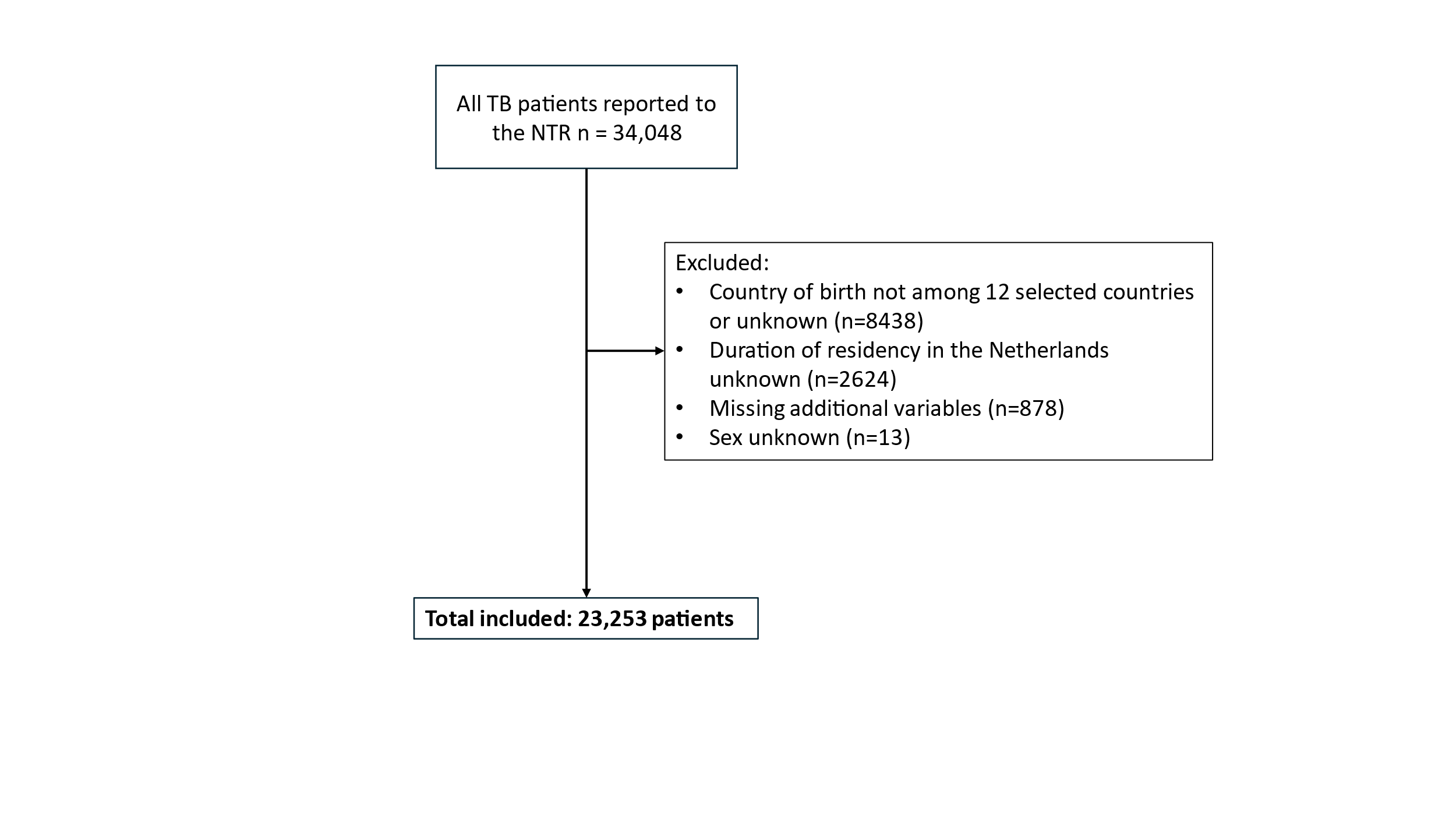


**Table S1: Numbers of patients with extrapulmonary tuberculosis (EPTB) and pulmonary tuberculosis (PTB) in age categories over the years of tuberculosis (TB) patients notified to the Netherlands, 1993-2022**

| Age | ≤ 14 (n) | | 15-24 (n) | | 25-34 (n) | | 35-44 (n) | | 45-64 (n) | | ≥65 (n) | |
| --- | --- | --- | --- | --- | --- | --- | --- | --- | --- | --- | --- | --- |
| Year | **EPTB** | **PTB** | **EPTB** | **PTB** | **EPTB** | **PTB** | **EPTB** | **PTB** | **EPTB** | **PTB** | **EPTB** | **PTB** |
| 1993 | 52 | 54 | 99 | 189 | 153 | 284 | 95 | 139 | 94 | 157 | 92 | 169 |
| 1994 | 79 | 33 | 147 | 174 | 229 | 293 | 120 | 134 | 134 | 157 | 125 | 175 |
| 1995 | 66 | 41 | 159 | 161 | 198 | 219 | 98 | 145 | 112 | 131 | 120 | 162 |
| 1996 | 74 | 32 | 145 | 151 | 228 | 215 | 107 | 125 | 116 | 163 | 123 | 170 |
| 1997 | 72 | 22 | 129 | 122 | 208 | 196 | 113 | 137 | 121 | 114 | 112 | 142 |
| 1998 | 73 | 20 | 134 | 132 | 189 | 168 | 107 | 112 | 109 | 100 | 113 | 105 |
| 1999 | 83 | 31 | 154 | 148 | 196 | 200 | 122 | 124 | 145 | 123 | 103 | 126 |
| 2000 | 69 | 30 | 119 | 160 | 228 | 174 | 113 | 131 | 114 | 120 | 82 | 103 |
| 2001 | 52 | 27 | 160 | 194 | 168 | 191 | 128 | 129 | 121 | 122 | 110 | 90 |
| 2002 | 61 | 23 | 139 | 164 | 181 | 200 | 109 | 125 | 129 | 125 | 77 | 82 |
| 2003 | 51 | 16 | 138 | 126 | 159 | 208 | 102 | 115 | 103 | 139 | 94 | 89 |
| 2004 | 60 | 22 | 114 | 124 | 150 | 148 | 103 | 111 | 135 | 127 | 92 | 106 |
| 2005 | 34 | 21 | 97 | 92 | 148 | 107 | 113 | 84 | 108 | 131 | 98 | 95 |
| 2006 | 35 | 16 | 77 | 85 | 126 | 106 | 85 | 99 | 126 | 101 | 90 | 84 |
| 2007 | 34 | 15 | 73 | 75 | 112 | 106 | 76 | 93 | 125 | 106 | 89 | 98 |
| 2008 | 35 | 15 | 82 | 92 | 129 | 109 | 104 | 85 | 98 | 115 | 74 | 74 |
| 2009 | 43 | 14 | 122 | 104 | 141 | 123 | 102 | 81 | 135 | 120 | 95 | 77 |
| 2010 | 23 | 11 | 95 | 73 | 171 | 102 | 110 | 84 | 132 | 104 | 83 | 80 |
| 2011 | 41 | 15 | 70 | 80 | 164 | 106 | 94 | 53 | 137 | 109 | 69 | 66 |
| 2012 | 30 | 20 | 76 | 52 | 144 | 112 | 90 | 64 | 123 | 95 | 93 | 57 |
| 2013 | 24 | 9 | 69 | 52 | 146 | 78 | 69 | 60 | 117 | 89 | 61 | 71 |
| 2014 | 40 | 8 | 68 | 51 | 113 | 82 | 75 | 58 | 104 | 91 | 69 | 55 |
| 2015 | 24 | 18 | 84 | 61 | 135 | 83 | 77 | 49 | 113 | 90 | 74 | 54 |
| 2016 | 35 | 14 | 83 | 86 | 135 | 80 | 83 | 50 | 114 | 75 | 71 | 61 |
| 2017 | 22 | 12 | 76 | 60 | 114 | 110 | 64 | 52 | 88 | 83 | 51 | 51 |
| 2018 | 16 | 5 | 81 | 86 | 118 | 79 | 62 | 63 | 83 | 89 | 65 | 48 |
| 2019 | 32 | 16 | 52 | 62 | 91 | 92 | 64 | 51 | 100 | 82 | 56 | 56 |
| 2020 | 16 | 3 | 45 | 46 | 109 | 61 | 82 | 29 | 74 | 66 | 51 | 39 |
| 2021 | 19 | 7 | 55 | 41 | 80 | 65 | 75 | 42 | 89 | 77 | 66 | 59 |
| 2022 | 15 | 6 | 48 | 53 | 80 | 60 | 61 | 60 | 76 | 93 | 45 | 38 |

**Table S2: Numbers of patients with extrapulmonary tuberculosis (EPTB) and pulmonary tuberculosis (PTB) and their countries of birth over the years of tuberculosis (TB) patients notified to the Netherlands, 1993-2022**

|  | Netherlands | | Eritrea | | Somalia | | India | | Ethiopia | | Pakistan | | Afghanistan | | Indonesia | | Morocco | | Poland | | Turkey | | Suriname | |
| --- | --- | --- | --- | --- | --- | --- | --- | --- | --- | --- | --- | --- | --- | --- | --- | --- | --- | --- | --- | --- | --- | --- | --- | --- |
|  | **EPTB** | **PTB** | **EPTB** | **PTB** | **EPTB** | **PTB** | **EPTB** | **PTB** | **EPTB** | **PTB** | **EPTB** | **PTB** | **EPTB** | **PTB** | **EPTB** | **PTB** | **EPTB** | **PTB** | **EPTB** | **PTB** | **EPTB** | **PTB** | **EPTB** | **PTB** |
| 1993 | 214 | 406 | 6 | 5 | 68 | 83 | 9 | 9 | 15 | 4 | 18 | 12 | 3 | 7 | 24 | 26 | 66 | 102 | 0 | 7 | 34 | 77 | 31 | 21 |
| 1994 | 314 | 469 | 6 | 3 | 110 | 74 | 12 | 7 | 15 | 8 | 17 | 13 | 5 | 3 | 23 | 13 | 86 | 95 | 0 | 4 | 29 | 48 | 39 | 34 |
| 1995 | 261 | 395 | 5 | 1 | 161 | 65 | 6 | 9 | 8 | 6 | 26 | 8 | 5 | 7 | 20 | 29 | 74 | 76 | 0 | 2 | 35 | 52 | 24 | 27 |
| 1996 | 300 | 426 | 4 | 1 | 145 | 59 | 9 | 11 | 11 | 4 | 18 | 9 | 7 | 5 | 28 | 27 | 65 | 91 | 0 | 3 | 26 | 44 | 28 | 23 |
| 1997 | 233 | 338 | 6 | 2 | 169 | 49 | 13 | 7 | 15 | 7 | 29 | 9 | 7 | 12 | 13 | 27 | 58 | 66 | 0 | 0 | 33 | 45 | 41 | 33 |
| 1998 | 216 | 281 | 2 | 1 | 131 | 51 | 14 | 5 | 11 | 11 | 23 | 6 | 22 | 12 | 27 | 20 | 69 | 61 | 1 | 1 | 22 | 37 | 28 | 22 |
| 1999 | 204 | 307 | 3 | 1 | 163 | 57 | 14 | 8 | 10 | 6 | 20 | 6 | 26 | 18 | 27 | 22 | 76 | 53 | 0 | 2 | 29 | 42 | 26 | 33 |
| 2000 | 176 | 259 | 8 | 4 | 135 | 64 | 17 | 4 | 15 | 11 | 21 | 7 | 24 | 11 | 24 | 24 | 58 | 67 | 0 | 1 | 19 | 38 | 30 | 26 |
| 2001 | 204 | 249 | 7 | 4 | 132 | 57 | 12 | 9 | 8 | 8 | 19 | 6 | 25 | 12 | 25 | 25 | 65 | 48 | 2 | 2 | 25 | 33 | 40 | 27 |
| 2002 | 167 | 250 | 12 | 5 | 114 | 44 | 17 | 9 | 8 | 9 | 18 | 8 | 16 | 19 | 29 | 28 | 48 | 70 | 2 | 0 | 29 | 45 | 25 | 27 |
| 2003 | 186 | 235 | 8 | 9 | 83 | 29 | 15 | 7 | 6 | 8 | 10 | 11 | 15 | 2 | 31 | 25 | 47 | 69 | 1 | 7 | 26 | 37 | 22 | 33 |
| 2004 | 207 | 237 | 11 | 12 | 55 | 24 | 14 | 4 | 9 | 10 | 18 | 6 | 12 | 11 | 45 | 21 | 57 | 60 | 0 | 1 | 22 | 34 | 43 | 18 |
| 2005 | 166 | 199 | 8 | 5 | 50 | 15 | 19 | 4 | 11 | 10 | 12 | 5 | 12 | 6 | 46 | 20 | 69 | 63 | 1 | 3 | 18 | 25 | 28 | 24 |
| 2006 | 172 | 196 | 5 | 5 | 45 | 18 | 10 | 7 | 3 | 5 | 5 | 4 | 14 | 2 | 31 | 16 | 59 | 35 | 0 | 5 | 17 | 24 | 32 | 25 |
| 2007 | 156 | 213 | 3 | 8 | 53 | 39 | 14 | 3 | 4 | 6 | 5 | 7 | 8 | 5 | 33 | 21 | 45 | 46 | 0 | 2 | 26 | 15 | 20 | 15 |
| 2008 | 134 | 173 | 4 | 4 | 107 | 45 | 19 | 6 | 4 | 2 | 7 | 4 | 12 | 8 | 22 | 17 | 64 | 36 | 2 | 10 | 16 | 21 | 24 | 20 |
| 2009 | 155 | 160 | 5 | 1 | 155 | 81 | 16 | 7 | 8 | 3 | 18 | 3 | 9 | 5 | 21 | 19 | 63 | 49 | 2 | 10 | 27 | 15 | 31 | 20 |
| 2010 | 132 | 147 | 7 | 10 | 154 | 47 | 22 | 2 | 8 | 4 | 13 | 4 | 18 | 7 | 22 | 18 | 58 | 34 | 1 | 10 | 21 | 15 | 26 | 17 |
| 2011 | 126 | 166 | 6 | 9 | 135 | 20 | 18 | 2 | 7 | 4 | 13 | 4 | 10 | 9 | 24 | 9 | 65 | 38 | 3 | 11 | 17 | 9 | 30 | 18 |
| 2012 | 125 | 131 | 8 | 1 | 127 | 42 | 20 | 9 | 12 | 1 | 12 | 3 | 10 | 5 | 26 | 18 | 52 | 33 | 1 | 13 | 21 | 9 | 27 | 20 |
| 2013 | 112 | 110 | 10 | 5 | 114 | 31 | 23 | 6 | 6 | 3 | 10 | 4 | 6 | 3 | 12 | 14 | 48 | 31 | 2 | 12 | 21 | 8 | 16 | 14 |
| 2014 | 119 | 94 | 27 | 26 | 87 | 19 | 22 | 8 | 3 | 3 | 12 | 1 | 8 | 6 | 15 | 22 | 49 | 30 | 0 | 10 | 13 | 12 | 19 | 13 |
| 2015 | 106 | 130 | 57 | 37 | 77 | 13 | 24 | 7 | 15 | 8 | 10 | 2 | 13 | 10 | 27 | 14 | 41 | 22 | 2 | 8 | 15 | 4 | 20 | 10 |
| 2016 | 110 | 105 | 73 | 45 | 64 | 30 | 23 | 5 | 21 | 11 | 9 | 5 | 12 | 8 | 21 | 19 | 39 | 26 | 4 | 3 | 16 | 8 | 19 | 14 |
| 2017 | 95 | 102 | 58 | 37 | 42 | 16 | 15 | 10 | 23 | 10 | 5 | 3 | 5 | 6 | 14 | 9 | 42 | 30 | 3 | 18 | 10 | 8 | 12 | 4 |
| 2018 | 83 | 96 | 83 | 48 | 33 | 10 | 32 | 13 | 24 | 5 | 4 | 0 | 10 | 2 | 19 | 11 | 34 | 31 | 4 | 19 | 10 | 12 | 8 | 6 |
| 2019 | 89 | 94 | 53 | 34 | 24 | 7 | 25 | 15 | 18 | 8 | 8 | 2 | 11 | 9 | 17 | 22 | 31 | 25 | 6 | 18 | 8 | 8 | 12 | 7 |
| 2020 | 81 | 86 | 49 | 25 | 14 | 6 | 35 | 7 | 12 | 3 | 6 | 3 | 7 | 5 | 21 | 8 | 41 | 22 | 4 | 12 | 7 | 5 | 8 | 3 |
| 2021 | 69 | 68 | 45 | 20 | 18 | 14 | 40 | 10 | 10 | 6 | 6 | 3 | 8 | 12 | 23 | 10 | 31 | 20 | 4 | 20 | 10 | 6 | 11 | 1 |
| 2022 | 58 | 67 | 37 | 33 | 25 | 11 | 19 | 5 | 17 | 8 | 5 | 4 | 7 | 4 | 10 | 9 | 35 | 24 | 10 | 24 | 9 | 5 | 4 | 1 |

**Table S3: Data of the Central Bureau of Statistics of population numbers (n) of both Dutch-born and born abroad, 1996-2021**

| Year | Netherlands | Afghanistan | Eritrea | Ethiopia | India | Indonesia | Morocco | Pakistan | Poland | Somalia | Suriname | Turkey |
| --- | --- | --- | --- | --- | --- | --- | --- | --- | --- | --- | --- | --- |
| 1996 | 12,995,174 | 7173 | 167 | 6565 | 6861 | 146,454 | 142,533 | 9821 | 14,112 | 19,801 | 179,873 | 169,034 |
| 1997 | 13,012,818 | 10740 | 214 | 6573 | 7207 | 144,233 | 145,604 | 9986 | 14,818 | 20,591 | 180,519 | 172,416 |
| 1998 | 13,033,792 | 14,601 | 271 | 6599 | 7700 | 142,843 | 149,469 | 10,095 | 15,648 | 21,027 | 182,467 | 175,229 |
| 1999 | 13,060,991 | 19,819 | 312 | 6703 | 7988 | 140,659 | 152,540 | 10,334 | 16,015 | 21,418 | 183,249 | 177,754 |
| 2000 | 13,088,648 | 24,254 | 383 | 6906 | 8265 | 138,936 | 155,669 | 10,649 | 17,028 | 21,705 | 184,737 | 181,595 |
| 2001 | 13,116,851 | 28,448 | 462 | 7149 | 8556 | 137,485 | 159,605 | 10,814 | 18,277 | 21,071 | 186,262 | 185,943 |
| 2002 | 13,140,336 | 30,936 | 553 | 7286 | 8695 | 135,561 | 163,280 | 10,921 | 19,716 | 19,546 | 187,279 | 190,219 |
| 2003 | 13,153,814 | 32,123 | 589 | 7233 | 8859 | 133,503 | 166,464 | 10,879 | 20,773 | 17,368 | 187,990 | 194,319 |
| 2004 | 13,169,880 | 32,394 | 645 | 7147 | 9029 | 131,216 | 168,400 | 10,651 | 24,566 | 15,083 | 188,367 | 195,678 |
| 2005 | 13,182,809 | 31,987 | 671 | 7038 | 9673 | 128,662 | 168,504 | 10,828 | 29,567 | 13,691 | 187,483 | 195,711 |
| 2006 | 13,186,595 | 31,330 | 716 | 7033 | 10,764 | 126,048 | 167,893 | 10,949 | 34,831 | 12,961 | 186,025 | 195,113 |
| 2007 | 13,187,586 | 30,939 | 811 | 7081 | 11,817 | 123,673 | 167,063 | 10,926 | 41,533 | 13,501 | 185,284 | 194,556 |
| 2008 | 13,189,983 | 30,707 | 898 | 7227 | 13,445 | 121,289 | 166,774 | 10,832 | 50,518 | 15,281 | 184,961 | 195,375 |
| 2009 | 13,198,081 | 31,060 | 1079 | 7457 | 14,284 | 118,963 | 167,305 | 11,041 | 57,496 | 19,803 | 185,089 | 196,385 |
| 2010 | 13,215,386 | 31,823 | 1299 | 7529 | 15,164 | 116,733 | 167,607 | 11,131 | 66,044 | 23,177 | 184,453 | 197,042 |
| 2011 | 13,228,780 | 32,579 | 1538 | 7748 | 16,500 | 114,558 | 168,214 | 11,333 | 77,642 | 24,638 | 183,752 | 197,107 |
| 2012 | 13,236,155 | 32,820 | 1705 | 7897 | 17,640 | 112,058 | 168,117 | 11,437 | 85,928 | 24,597 | 182,342 | 196,203 |
| 2013 | 13,236,494 | 33,085 | 2103 | 8013 | 19,175 | 109,788 | 168,320 | 11,672 | 95,617 | 26,502 | 180,863 | 194,759 |
| 2014 | 13,234,545 | 33,058 | 4141 | 8935 | 21,273 | 107,541 | 168,451 | 11,760 | 107,891 | 27,275 | 179,236 | 192,311 |
| 2015 | 13,235,405 | 33,030 | 7386 | 11,318 | 23,991 | 105,235 | 168,336 | 12,093 | 117,269 | 26,803 | 177,720 | 190,621 |
| 2016 | 13,226,829 | 34,694 | 10,454 | 14,086 | 27,580 | 103,245 | 168,536 | 12,458 | 125,978 | 26,094 | 176,801 | 190,331 |
| 2017 | 13,218,754 | 34,993 | 13,357 | 15,529 | 32,285 | 100,922 | 169,018 | 12,944 | 134,999 | 25,645 | 176,412 | 191,513 |
| 2018 | 13,209,225 | 35,490 | 15,868 | 16,866 | 38,194 | 98,772 | 170,357 | 13,563 | 144,580 | 25,212 | 176,564 | 193,698 |
| 2019 | 13,196,025 | 35,837 | 18,200 | 17,814 | 45,147 | 96,719 | 172,040 | 14,373 | 154,588 | 24,951 | 176,963 | 197,446 |
| 2020 | 13,186,880 | 36,305 | 19,807 | 18,458 | 46,191 | 93,636 | 172,542 | 14,829 | 163,522 | 24,799 | 176,571 | 199,402 |
| 2021 | 13,169,507 | 38,388 | 21,042 | 19,119 | 52,102 | 91,556 | 173,279 | 15,715 | 172,820 | 24,693 | 176,127 | 204,249 |

**Table S4: Incidences per 100000 of tuberculosis (TB) patients per country of birth, 1996-2021**

| Incidences total TB | Netherlands | Somalia | Morocco | Turkey | Suriname | Indonesia | Pakistan | Ethiopia | India | Eritrea | Afghanistan | Poland |
| --- | --- | --- | --- | --- | --- | --- | --- | --- | --- | --- | --- | --- |
| 1996 | 5.6 | 1030.3 | 109.4 | 41.4 | 28.4 | 37.6 | 274.9 | 228.5 | 291.5 | 2994.0 | 167.2 | 21.3 |
| 1997 | 4.4 | 1058.1 | 85.2 | 45.2 | 41.0 | 27.7 | 380.5 | 334.7 | 277.5 | 3738.3 | 176.9 | 0.0 |
| 1998 | 3.8 | 849.8 | 86.9 | 33.7 | 27.4 | 32.9 | 287.3 | 333.4 | 246.8 | 1107.0 | 232.9 | 12.8 |
| 1999 | 3.9 | 1027.2 | 84.6 | 39.9 | 32.2 | 34.8 | 251.6 | 238.7 | 275.4 | 1282.1 | 222.0 | 12.5 |
| 2000 | 3.3 | 916.8 | 80.3 | 31.4 | 30.3 | 34.5 | 262.9 | 376.5 | 254.1 | 3133.2 | 144.3 | 59.0 |
| 2001 | 3.5 | 897.0 | 69.2 | 31.2 | 36.0 | 36.4 | 231.2 | 223.8 | 245.4 | 2381.0 | 130.0 | 21.9 |
| 2002 | 3.2 | 808.3 | 72.3 | 38.9 | 27.8 | 42.0 | 238.1 | 233.3 | 299.0 | 3074.1 | 113.1 | 10.1 |
| 2003 | 3.2 | 644.9 | 69.7 | 32.4 | 29.3 | 41.9 | 193.0 | 193.6 | 248.3 | 2886.2 | 52.9 | 38.5 |
| 2004 | 3.4 | 523.8 | 69.4 | 28.6 | 32.4 | 50.3 | 225.3 | 265.8 | 199.4 | 3565.9 | 71.0 | 4.1 |
| 2005 | 2.8 | 474.8 | 78.3 | 22.0 | 27.7 | 51.3 | 157.0 | 298.6 | 237.8 | 1937.4 | 56.2 | 13.5 |
| 2006 | 2.8 | 486.1 | 56.0 | 21.0 | 30.6 | 37.3 | 82.2 | 113.7 | 157.9 | 1396.6 | 51.1 | 14.4 |
| 2007 | 2.8 | 681.4 | 54.5 | 21.1 | 18.9 | 43.7 | 109.8 | 141.2 | 143.9 | 1356.4 | 42.0 | 4.8 |
| 2008 | 2.3 | 994.7 | 60.0 | 18.9 | 23.8 | 32.2 | 101.6 | 83.0 | 185.9 | 890.9 | 65.1 | 23.8 |
| 2009 | 2.4 | 1191.7 | 66.9 | 21.4 | 27.6 | 33.6 | 190.2 | 147.5 | 161.0 | 556.1 | 45.1 | 20.9 |
| 2010 | 2.1 | 867.2 | 54.9 | 18.3 | 23.3 | 34.3 | 152.7 | 159.4 | 158.3 | 1308.7 | 78.6 | 16.7 |
| 2011 | 2.2 | 629.1 | 61.2 | 13.2 | 26.1 | 28.8 | 150.0 | 142.0 | 121.2 | 975.3 | 58.3 | 18.0 |
| 2012 | 1.9 | 687.1 | 50.6 | 15.3 | 25.8 | 39.3 | 131.2 | 164.6 | 164.4 | 527.9 | 46.0 | 16.3 |
| 2013 | 1.7 | 547.1 | 46.9 | 14.9 | 16.6 | 23.4 | 119.9 | 112.3 | 151.2 | 713.3 | 27.2 | 14.6 |
| 2014 | 1.6 | 388.6 | 46.9 | 13.0 | 17.9 | 34.4 | 110.5 | 67.1 | 141.0 | 1279.9 | 42.3 | 9.3 |
| 2015 | 1.8 | 335.8 | 37.4 | 10.0 | 16.9 | 39.0 | 99.2 | 203.2 | 129.2 | 1272.7 | 69.6 | 8.5 |
| 2016 | 1.6 | 360.2 | 38.6 | 12.6 | 18.7 | 38.7 | 112.4 | 227.2 | 101.5 | 1128.8 | 57.6 | 5.6 |
| 2017 | 1.5 | 226.2 | 42.6 | 9.4 | 9.1 | 22.8 | 61.8 | 212.5 | 77.4 | 711.2 | 31.4 | 15.6 |
| 2018 | 1.4 | 170.6 | 38.1 | 11.4 | 7.9 | 30.4 | 29.5 | 171.9 | 117.8 | 825.6 | 33.8 | 15.9 |
| 2019 | 1.4 | 124.2 | 32.6 | 8.1 | 10.7 | 40.3 | 69.5 | 146.0 | 88.6 | 478.0 | 55.8 | 15.5 |
| 2020 | 1.3 | 80.6 | 36.5 | 5.5 | 6.2 | 31.0 | 60.7 | 81.3 | 90.9 | 373.6 | 33.0 | 9.8 |
| 2021 | 1.1 | 129.6 | 29.4 | 7.8 | 6.8 | 36.0 | 57.2 | 83.7 | 96.0 | 308.9 | 52.1 | 13.9 |

**Table S5: Incidences of patients with extrapulmonary tuberculosis (EPTB) per country of birth calculated per 100,000, 1996-2021**

| Incidences  EPTB | Netherlands | Somalia | Morocco | Turkey | Suriname | Indonesia | Pakistan | Ethiopia | India | Eritrea | Afghanistan | Poland |
| --- | --- | --- | --- | --- | --- | --- | --- | --- | --- | --- | --- | --- |
| 1996 | 2.3 | 732.3 | 45.6 | 15.4 | 15.6 | 19.1 | 183.3 | 167.6 | 131.7 | 2395.2 | 97.6 | 0 |
| 1997 | 1.8 | 820.7 | 39.8 | 19.1 | 23.8 | 8.9 | 290.4 | 228.2 | 180.4 | 2803.7 | 65.2 | 0 |
| 1998 | 1.7 | 623.0 | 46.2 | 12.6 | 15.3 | 18.9 | 227.8 | 166.7 | 181.8 | 738.0 | 150.7 | 6.4 |
| 1999 | 1.6 | 761.0 | 49.8 | 16.3 | 14.2 | 19.2 | 193.5 | 149.2 | 175.3 | 961.5 | 131.2 | 0 |
| 2000 | 1.3 | 622.0 | 37.3 | 10.5 | 16.2 | 17.3 | 197.2 | 217.2 | 205.7 | 2088.8 | 99.0 | 0 |
| 2001 | 1.6 | 626.5 | 40.7 | 13.4 | 21.5 | 18.2 | 175.7 | 111.9 | 140.3 | 1515.2 | 87.9 | 10.9 |
| 2002 | 1.3 | 583.2 | 29.4 | 15.2 | 13.3 | 21.4 | 164.8 | 109.8 | 195.5 | 2170.0 | 51.7 | 10.1 |
| 2003 | 1.4 | 477.9 | 28.2 | 13.4 | 11.7 | 23.2 | 91.9 | 83.0 | 169.3 | 1358.2 | 46.7 | 4.8 |
| 2004 | 1.6 | 364.6 | 33.8 | 11.2 | 22.8 | 34.3 | 169.0 | 125.9 | 155.1 | 1705.4 | 37.0 | 0 |
| 2005 | 1.3 | 365.2 | 40.9 | 9.2 | 14.9 | 35.6 | 110.8 | 156.3 | 196.4 | 1192.3 | 37.5 | 3.4 |
| 2006 | 1.3 | 347.2 | 35.1 | 8.7 | 17.2 | 24.6 | 45.7 | 42.7 | 92.9 | 698.3 | 44.7 | 0 |
| 2007 | 1.2 | 392.6 | 26.9 | 13.4 | 10.8 | 26.7 | 45.8 | 56.5 | 118.5 | 369.9 | 25.9 | 0 |
| 2008 | 1.0 | 700.2 | 38.4 | 8.2 | 13.0 | 18.1 | 64.6 | 55.3 | 141.3 | 445.4 | 39.1 | 4.0 |
| 2009 | 1.2 | 782.7 | 37.7 | 13.7 | 16.7 | 17.7 | 163.0 | 107.3 | 112.0 | 463.4 | 29.0 | 3.5 |
| 2010 | 1.0 | 664.5 | 34.6 | 10.7 | 14.1 | 18.8 | 116.8 | 106.3 | 145.1 | 538.9 | 56.6 | 1.5 |
| 2011 | 1.0 | 547.9 | 38.6 | 8.6 | 16.3 | 21.0 | 114.7 | 90.3 | 109.1 | 390.1 | 30.7 | 3.9 |
| 2012 | 0.9 | 516.3 | 30.9 | 10.7 | 14.8 | 23.2 | 104.9 | 152.0 | 113.4 | 469.2 | 30.5 | 1.2 |
| 2013 | 0.8 | 430.2 | 28.5 | 10.8 | 8.8 | 10.9 | 85.7 | 74.9 | 119.9 | 475.5 | 18.1 | 2.1 |
| 2014 | 0.9 | 319.0 | 29.1 | 6.8 | 10.6 | 13.9 | 102.0 | 33.6 | 103.4 | 652.0 | 24.2 | 0 |
| 2015 | 0.8 | 287.3 | 24.4 | 7.9 | 11.3 | 25.7 | 82.7 | 132.5 | 100.0 | 771.7 | 39.4 | 1.7 |
| 2016 | 0.8 | 245.3 | 23.1 | 8.4 | 10.7 | 20.3 | 72.2 | 149.1 | 83.4 | 698.3 | 34.6 | 3.2 |
| 2017 | 0.7 | 163.8 | 24.8 | 5.2 | 6.8 | 13.9 | 38.6 | 148.1 | 46.5 | 434.2 | 14.3 | 2.2 |
| 2018 | 0.6 | 130.9 | 20.0 | 5.2 | 4.5 | 19.2 | 29.4 | 142.3 | 83.8 | 523.1 | 28.2 | 2.8 |
| 2019 | 0.7 | 96.2 | 18.0 | 4.1 | 6.8 | 17.6 | 55.7 | 101.0 | 55.4 | 291.2 | 30.7 | 3.9 |
| 2020 | 0.6 | 56.5 | 23.8 | 3.5 | 4.5 | 22.4 | 40.5 | 65.0 | 75.8 | 247.3 | 19.3 | 2.4 |
| 2021 | 0.5 | 72.9 | 17.9 | 4.9 | 6.2 | 25.1 | 38.2 | 52.3 | 78.8 | 213.9 | 20.8 | 2.3 |

**Table S6: Patients with extrapulmonary tuberculosis (EPTB) and pulmonary tuberculosis (PTB) to sex per country of birth notified to the Netherlands, 1993-2023**

| Country of birth | Female | | | | Male | | | |
| --- | --- | --- | --- | --- | --- | --- | --- | --- |
|  | EPTB | | PTB | | EPTB | | PTB | |
|  | n | % | n | % | n | % | n | % |
| Netherlands | 2274 | 51.1 | 2170 | 48.9 | 2762 | 42.4 | 3750 | 57.6 |
| Eritrea | 242 | 68.0 | 114 | 32.0 | 381 | 57.6 | 280 | 42.4 |
| Somalia | 1342 | 78.7 | 363 | 21.3 | 1465 | 66.5 | 739 | 33.5 |
| India | 204 | 73.4 | 74 | 26.6 | 348 | 71.9 | 136 | 28.1 |
| Ethiopia | 151 | 70.9 | 62 | 29.1 | 191 | 60.4 | 125 | 39.6 |
| Pakistan | 167 | 75.6 | 54 | 24.4 | 232 | 68.6 | 106 | 31.4 |
| Afghanistan | 191 | 61.0 | 122 | 39.0 | 155 | 58.5 | 110 | 41.5 |
| Indonesia | 473 | 60.2 | 312 | 39.8 | 254 | 51.1 | 243 | 48.9 |
| Morocco | 843 | 65.1 | 453 | 34.9 | 816 | 45.5 | 976 | 54.5 |
| Poland | 17 | 20.5 | 66 | 79.5 | 39 | 18.6 | 171 | 81.4 |
| Turkey | 327 | 63.1 | 191 | 36.9 | 292 | 35.1 | 540 | 64.9 |
| Suriname | 335 | 69.5 | 147 | 30.5 | 401 | 50.3 | 396 | 49.7 |
